# An Important Association With Lower Gastrointestinal Bleed: A Case of Heyde Syndrome

**DOI:** 10.7759/cureus.26232

**Published:** 2022-06-23

**Authors:** Anass Dweik, Ola Al-Jabory, Waqas Rasheed, Muhammad Ali Anees, Noor Dweik

**Affiliations:** 1 Internal Medicine, Texas Tech University Health Sciences Center, Amarillo, USA; 2 Internal Medicine/Resident, Texas Tech University Health Sciences Center, Amarillo, USA

**Keywords:** acquired vwf factor deficiency, transcatheter aortic replacement, intestinal angiodysplasia, aortic stenosis (as), gastrointestinal bleed, heyde syndrome

## Abstract

The association between aortic stenosis and angiodysplastic gastrointestinal bleed is known as Heyde syndrome. It was first described in 1958 and has since received further medical attention. We present a case of an 86-year-old lady with a history of severe aortic stenosis that was admitted with gastrointestinal bleeding secondary to colonic angiodysplasia. A review of the literature showed mixed opinions with respect to the idea of causation versus coincidence; both theories are valid. However, studies that supported causation had a bigger study population and overall seem to be more plausible.

## Introduction

First described in 1958, Heyde syndrome is a condition that is defined by a triad of aortic stenosis (AS), gastrointestinal (GI) bleeding from angiodysplasia, and acquired Von Willebrand syndrome [1][2]. Since the time it was first described, there have been multiple case reports and reviews of literature about Heyde syndrome. Nevertheless, it continues to be a diagnosis that is often missed in clinical practice. Hence, we are presenting a case of Heyde syndrome in an 86-year-old female that was admitted for a left femur fracture secondary to severe hematochezia from colonic angiodysplasia that led to the patient losing balance and falling at home. The aim of this case report is to bring forward the importance of keeping Heyde syndrome among the differential diagnosis when dealing with elderly patients presenting with gastrointestinal (GI) bleeding and AS murmur.

## Case presentation

An 86-year-old lady presented to the emergency department following an episode of fall at home. As per the patient, she was working around her house when she suddenly slipped on her own blood and fell on her bottom. She ended up having severe hip pain for which she decided to seek medical attention.

Upon further history, the patient stated that she has been having frequent episodes of hematochezia over the last three months that would often form a pool of blood below her as she had difficulties ambulating. She has been in and out of multiple hospitals for blood transfusions in the recent past. She denied epistaxis, hemoptysis, hematemesis, or hematuria. She also denied using aspirin or nonsteroidal anti-inflammatory drugs (NSAIDs). Her medical history was significant for atrial fibrillation and aortic stenosis with an aortic valve area of < 1 cm as evident on an echocardiogram done three weeks prior to admission. She was prescribed apixaban for her arrhythmia; however, she was not taking the medication due to her ongoing hematochezia and blood loss. She was told that her aortic stenosis is severe and was scheduled to get a transcatheter aortic valve replacement (TAVR) in a month’s time.

Initial vitals included a heart rate of 90 beats/minute and blood pressure of 180/87 mmHg. She was significantly pale and in moderate distress due to her hip pain. Cardiovascular examination was significant for a grade 4/6 systolic murmur at the right second intercostal space with radiation to the carotids. A lower limb examination revealed an externally rotated left lower limb with a restricted range of motion. A digital rectal examination showed fresh right red blood with no evidence of hemorrhoids. Laboratory studies showed hemoglobin of 7 g/dL, platelet count of 134,000/ml, prothrombin time of 11.6 seconds, partial thromboplastin time of 26 seconds, and international normalized ratio of 1.12 blood urea nitrogen (BUN) of 16 mg/dL, and creatinine of 0.7 mg/dL. A complete left hip x-ray revealed an acute left femur intertrochanteric fracture for which orthopedics were consulted; however, the decision was made to postpone surgery until the patient’s gastrointestinal (GI) bleed resolves. She had a colonoscopy is done that showed a large amount of old blood throughout the colon, mostly in the dependent portion of the colon. Arteriovenous (AV) malformations were noted at the proximal ascending colon that was clipped. A nuclear medicine gastrointestinal blood loss imaging showed evidence for extravasation of labeled red blood cells into the bowel lumen at the lateral right lower abdomen/upper pelvis favoring an ascending colon bleeding site. The patient was treated conservatively with blood transfusions, fluids, and analgesics. She was later taken for open reduction internal fixation (ORIF) and her hip was stabilized. Following her surgery, cardiology was consulted and recommended conservative therapy until the patient gets her already scheduled TAVR procedure done as an outpatient at a tertiary center. The patient was monitored closely thereafter and was eventually discharged to a skilled nursing facility for physical therapy given her fracture and was advised thoroughly to get to her TAVR procedure appointment.

## Discussion

The association between AS and angiodysplastic GI bleeding is known as Heyde syndrome. The condition is named after Dr. Heyde who first described the condition in 1958 after noticing a group of 10 patients between sixty and eighty years of age that had calcific aortic stenosis with massive GI bleed [1]. Since that time, further studies have been able to describe the same association, and the condition has been well established in the literature. Nevertheless, there has been some controversy if the association is coincidental or a causal relationship.

The pathogenesis is thought to be related to a relative acquired type 2A von Willebrand factor (vWF) deficiency secondary to aortic stenosis [3]. vWF is a complex disulfide-linked protein that has a size range of 860,000 to over 10 million daltons with a complex multimeric structure. Its structure helps facilitate platelet adhesion and aggregation to the subendothelium [4]. Acquired type 2A vWF deficiency is a condition where the largest vWF multimers are deficient [5]. It is thought proteolysis of vWF occurs as it passes through a stenotic valve that leads to exposure of the bond between amino acids 842 and 843 which happens to affect a specific vWF protease. Thus, proteolysis of the highest molecular-weight multimers of vWF occurs hindering the function of the most effective platelet-mediated hemostasis in a state of high shear stress [6][7][8]. Two studies have shown that patients with type 2A vWF deficiency secondary to either congenital or acquired cardiac disease have their vWF multimer pattern reverting to the normal following operative intervention of their cardiac disease [9][10]. Despite that, the 2020 ACC/AHA guideline for the management of patients with valvular heart disease only counts cardiac symptoms in the evaluation of valve replacement [11]. Endoscopy is currently the modality of choice to diagnose and treat intestinal angiodysplasia. Argon Plasma Coagulation (APC) is the best endoscopy-guided therapy while octreotide and thalidomide are second-line pharmacological therapy in a selected group of patients. Nevertheless, the recurrence rate of APC and pharmacological therapy is high but is considered a good alternative in patients who do not match the criteria for aortic valve replacement/repair or who are at high risk for surgical intervention [12].

## Conclusions

In conclusion, Heyde syndrome should be within the differential diagnosis when evaluating an elderly patient that presents with GI bleed. A cardiac exam along with an echocardiogram should be considered as these patients seem to have improvement in their GI bleed following AS repair. Furthermore, it would always help to have a multidisciplinary team including cardiology, gastroenterology, and hematology when suspecting a case of heyde syndrome to come up with a definitive treatment plan and to avoid multiple readmissions. 
